# The British and Foreign Medico-Chirurgical Review

**Published:** 1858-01

**Authors:** 


*The British and Foreign Medico-Chirurgical Review.*—The October number of this excellent quarterly is full of interesting matter. The article on Resections of the Joints is a capital resumé of the present practice of surgical art. Equally important is the consideration of the utility of vaccination, a paper in which the recent contributions of medical science to the determination of that vital question of sanitary police are fully discussed, and the opinion maintained that vaccination has most materially diminished the mortality from small-pox. Among the physiological articles, we would call special attention to that on the Physiology of the Iris, and that on Parthenogenesis in Moths and Bees. Among the original communications we mention as worthy of particular notice, Mr. Rainey's paper on the Formation of the Skeletons of Animals and other hard structures formed in connection with living tissue.

We cordially recommend this Review to our readers, as containing a very full and satisfactory account of what is doing in medical science. Messrs. S. S. & W. Wood, 389 Broadway, are the American publishers.

